# Methyl 3-[(1-adamantylcarbon­yloxy)amino­carbon­yl]propanoate

**DOI:** 10.1107/S1600536809024210

**Published:** 2009-07-04

**Authors:** Joe Liu, Jack K. Clegg, Rachel Codd

**Affiliations:** a School of Medical Sciences (Pharmacology) and Bosch Institute, D06, The University of Sydney, New South Wales 2006, Australia; b Centre for Heavy Metals Research, School of Chemistry, F11, University of Sydney, New South Wales 2006, Australia

## Abstract

In the title compound, C_16_H_23_NO_5_, the H—N—O—C torsion angle is 98.6 (1)°, which is of a similar magnitude to other *N*,*O*-diacyl­hydroxy­lamines. The N—O distance is 1.4029 (14) Å, which is similar to the N—O distance in other *N*,*O*-diacyl­hydroxy­lamines. In the crystal, intermolecular N—H⋯O hydrogen bonds generate chains of molecules.

## Related literature

For the biological activity of compounds related to *N*,*O*-diacyl­hydroxy­lamines, see: Pelto & Pratt (2008 ▶). For linear *N*,*O*-diacyl­hydroxy­lamines, see: Göttlicher & Ochsenreiter (1974 ▶); Schraml *et al.* (2004 ▶); Baert *et al.* (1984 ▶); Masui *et al.* (1983 ▶); Grassi *et al.* (2002 ▶); Buscemi *et al.* (2006 ▶). For cyclic *N*,*O*-diacyl­hydroxy­lamines, see: Kongprakaiwoot *et al.* (2008 ▶). For a precursor of the title compound, see: Liu *et al.* (2009 ▶).
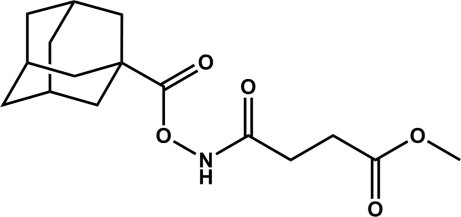


## Experimental

### 

#### Crystal data

C_16_H_23_NO_5_
*M* *_r_* = 309.35
Orthorhombic, 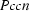

*a* = 15.7837 (5) Å
*b* = 21.0715 (7) Å
*c* = 9.5341 (3) Å
*V* = 3170.91 (18) Å^3^
*Z* = 8
Mo *K*α radiation
μ = 0.10 mm^−1^
*T* = 150 K
0.30 × 0.20 × 0.15 mm

#### Data collection

Bruker APEXII-FR591 diffractometer
Absorption correction: multi-scan (*SADABS*; Sheldrick, 2007 ▶) *T*_min_ = 0.851, *T*_max_ = 0.981
22952 measured reflections
4586 independent reflections
2890 reflections with *I* > 2σ(*I*)
*R*_int_ = 0.036

#### Refinement

*R*[*F* ^2^ > 2σ(*F* ^2^)] = 0.048
*wR*(*F* ^2^) = 0.132
*S* = 1.04
4586 reflections
200 parameters
H-atom parameters constrained
Δρ_max_ = 0.25 e Å^−3^
Δρ_min_ = −0.26 e Å^−3^

### 

Data collection: *APEX2* (Bruker, 2003 ▶); cell refinement: *SAINT* (Bruker, 2003 ▶); data reduction: *SAINT* and *XPREP* (Bruker, 2003 ▶); program(s) used to solve structure: *SIR97* (Altomare *et al.*, 1999 ▶); program(s) used to refine structure: *SHELXL97* (Sheldrick, 2008 ▶); molecular graphics: *ORTEP-3* (Farrugia, 1997 ▶), *WinGX32* (Farrugia, 1999 ▶), *POV-RAY* (Cason, 2002 ▶) and *WebLab ViewerPro* (Molecular Simulations, 2000 ▶); software used to prepare material for publication: *enCIFer* (Allen *et al.*, 2004 ▶).

## Supplementary Material

Crystal structure: contains datablocks global, I. DOI: 10.1107/S1600536809024210/bg2266sup1.cif

Structure factors: contains datablocks I. DOI: 10.1107/S1600536809024210/bg2266Isup2.hkl

Additional supplementary materials:  crystallographic information; 3D view; checkCIF report

## Figures and Tables

**Table 1:** Hydrogen-bond geometry (Å, °)

*D*—H⋯*A*	*D*—H	H⋯*A*	*D*⋯*A*	*D*—H⋯*A*
N1—H1⋯O3^i^	0.88	1.87	2.7250 (19)	165

Symmetry code: (i) 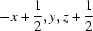.

**Fig. 1.**
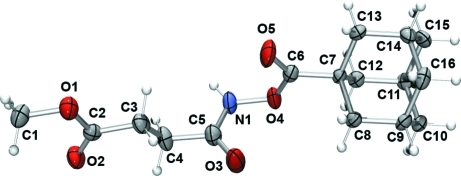
ORTEP representation of I shown with 50% probability ellipsoids.

**Fig. 2.**
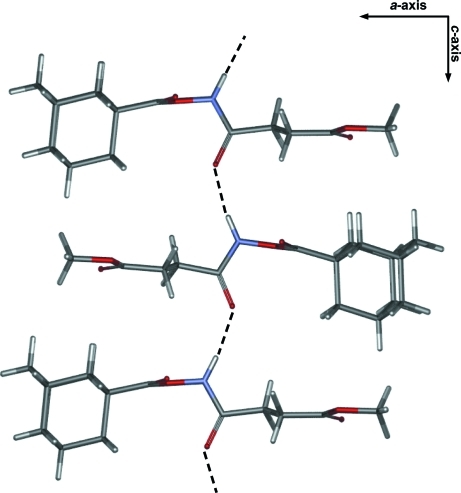
A schematic representation of the one dimenisonal polymer formed through hydrogen bonding interactions in I. Dashed lines indicate hydrogen bonds.

## supplementary crystallographic information

### Comment

*O*-Adamantanecarboxoyl-*N*-4-methoxy-4-oxobutanoyl-hydroxylamine (I) (Fig 1.) was prepared in our laboratory as part of our program in understanding resonance and tautomerism in biologically relevant molecules such as *N,O*-diacylhydroxylamines and hydroxamic acids. The torsion angle defined by H—N—O—C in each of *N,O*-diacetylhydroxylamine (-99.0 (1) °), *N*-acetyl-*O*-benzoylhydroxylamine (-101.3 (1) °), and *N*-benzoyl-*O*-acetylhydroxylamine (-94.1 (1) °) is negative, which is distinct from the analogous angle in *N*,*O*-dibenzoylhydroxylamine determined by the same group, which is positive (99.7 (1) °) (Schraml *et al.*, 2004). The positive torsion angle defined by H1—N1—O4—C6 in I is 98.6 (1) °, which is akin to *N*,*O*-dibenzoylhydroxylamine. The N—O distance in I (N1—O4 = 1.4029 (14) Å) is similar to the N—O distance in other *N*,*O*-diacylhydroxylamines as cited above. Intermolecular hydrogen bonds exist in I between respective amide groups, with H1···O3 = 1.87 Å (Table 1) forming an infinite one-dimensional polymer extending along the *c*-axis (Fig 2.).

### Experimental

*O*-Adamantanecarboxoyl-*N*-4-methoxy-4-oxobutanoyl-hydroxylamine (I) was isolated from a methanol solution (14 ml) containing adamantane-1-carboxylate-2,5-pyrrolidinedione (0.25 g, 0.89 mmol) (Liu, *et al.*, 2009) and NaOH (0.018 g, 0.45 mmol). The product was dried *in vacuo*; colourless crystals of I appeared after approximately 1 month from a 4.5 mg m*L*^-1^ solution of I in ethanol:water (7:3).

### Refinement

C and N bound-H (atoms were included in idealized positions and refined using a riding-model approximation, with C—H bond lengths fixed at 1.00 Å, 0.99 Å, 0.98 Å for methine, methylene and methyl H atoms respectively. N—H bond lengths fixed at 0.88 Å. *U*_iso_(H) values were fixed at 1.2*U*_eq_ of the parent atoms for all H atoms except methyl H atoms for which 1.5*U*_eq_ of the parent atoms was used.

### Crystal data

**Table:**

C_16_H_23_NO_5_	*F*(000) = 1328
*M**_r_* = 309.35	*D*_x_ = 1.296 Mg m^−^^3^
Orthorhombic, *P**c**c**n*	Mo *K*α radiation, λ = 0.71073 Å
Hall symbol: -P 2ab 2ac	Cell parameters from 3869 reflections
*a* = 15.7837 (5) Å	θ = 2.8–30.0°
*b* = 21.0715 (7) Å	µ = 0.10 mm^−^^1^
*c* = 9.5341 (3) Å	*T* = 150 K
*V* = 3170.91 (18) Å^3^	Block, colourless
*Z* = 8	0.30 × 0.20 × 0.15 mm

### Data collection

**Table:**

Bruker APEXII-FR591 diffractometer	4586 independent reflections
Radiation source: rotating anode	2890 reflections with *I* > 2σ(*I*)
graphite	*R*_int_ = 0.036
ω+φ scans	θ_max_ = 30.0°, θ_min_ = 3.2°
Absorption correction: multi-scan (*SADABS*; Sheldrick, 2007)	*h* = −22→22
*T*_min_ = 0.851, *T*_max_ = 0.981	*k* = −26→29
22952 measured reflections	*l* = −13→11

### Refinement

**Table:**

Refinement on *F*^2^	Primary atom site location: structure-invariant direct methods
Least-squares matrix: full	Secondary atom site location: difference Fourier map
*R*[*F*^2^ > 2σ(*F*^2^)] = 0.048	Hydrogen site location: inferred from neighbouring sites
*wR*(*F*^2^) = 0.132	H-atom parameters constrained
*S* = 1.04	*w* = 1/[σ^2^(*F*_o_^2^) + (0.0599*P*)^2^ + 0.4178*P*] where *P* = (*F*_o_^2^ + 2*F*_c_^2^)/3
4586 reflections	(Δ/σ)_max_ < 0.001
200 parameters	Δρ_max_ = 0.25 e Å^−^^3^
0 restraints	Δρ_min_ = −0.25 e Å^−^^3^

### Special details

**Table:**

Experimental. The crystal was coated in Exxon Paratone N hydrocarbon oil and mounted on a thin mohair fibre attached to a copper pin. Upon mounting on the diffractometer, the crystal was quenched to 150(K) under a cold nitrogen gas stream supplied by an Oxford Cryosystems Cryostream and data were collected at this temperature.
Geometry. All e.s.d.'s (except the e.s.d. in the dihedral angle between two l.s. planes) are estimated using the full covariance matrix. The cell e.s.d.'s are taken into account individually in the estimation of e.s.d.'s in distances, angles and torsion angles; correlations between e.s.d.'s in cell parameters are only used when they are defined by crystal symmetry. An approximate (isotropic) treatment of cell e.s.d.'s is used for estimating e.s.d.'s involving l.s. planes.
Refinement. Refinement of *F*^2^ against ALL reflections. The weighted *R*-factor *wR* and goodness of fit *S* are based on *F*^2^, conventional *R*-factors *R* are based on *F*, with *F* set to zero for negative *F*^2^. The threshold expression of *F*^2^ > σ(*F*^2^) is used only for calculating *R*-factors(gt) *etc*. and is not relevant to the choice of reflections for refinement. *R*-factors based on *F*^2^ are statistically about twice as large as those based on *F*, and *R*- factors based on ALL data will be even larger.

### Fractional atomic coordinates and isotropic or equivalent isotropic displacement parameters (Å^2^)

**Table:**

	*x*	*y*	*z*	*U*_iso_*/*U*_eq_	
C1	−0.10511 (9)	0.03188 (7)	0.34190 (19)	0.0397 (4)	
H1A	−0.1286	0.0521	0.4259	0.060*	
H1B	−0.1195	−0.0134	0.3422	0.060*	
H1C	−0.1289	0.0520	0.2580	0.060*	
C2	0.01542 (9)	0.09834 (6)	0.33007 (15)	0.0282 (3)	
C3	0.11059 (9)	0.09992 (7)	0.32346 (18)	0.0347 (3)	
H3A	0.1293	0.0852	0.2299	0.042*	
H3B	0.1339	0.0704	0.3944	0.042*	
C4	0.14536 (9)	0.16607 (7)	0.34972 (19)	0.0395 (4)	
H4A	0.1174	0.1965	0.2855	0.047*	
H4B	0.1321	0.1790	0.4471	0.047*	
C5	0.24014 (10)	0.16868 (7)	0.32743 (19)	0.0381 (4)	
C6	0.41331 (9)	0.12429 (6)	0.41915 (16)	0.0285 (3)	
C7	0.50576 (8)	0.13370 (5)	0.38462 (14)	0.0217 (3)	
C8	0.51335 (8)	0.13881 (6)	0.22281 (15)	0.0264 (3)	
H8A	0.4889	0.1005	0.1782	0.032*	
H8B	0.4816	0.1763	0.1890	0.032*	
C9	0.60700 (8)	0.14499 (6)	0.18337 (15)	0.0272 (3)	
H9	0.6124	0.1480	0.0791	0.033*	
C10	0.64442 (8)	0.20472 (6)	0.25077 (15)	0.0277 (3)	
H10A	0.7048	0.2090	0.2240	0.033*	
H10B	0.6137	0.2427	0.2167	0.033*	
C11	0.63684 (8)	0.20033 (6)	0.40991 (15)	0.0271 (3)	
H11	0.6612	0.2395	0.4533	0.032*	
C12	0.54299 (8)	0.19452 (6)	0.45073 (16)	0.0269 (3)	
H12A	0.5114	0.2321	0.4169	0.032*	
H12B	0.5375	0.1926	0.5541	0.032*	
C13	0.55512 (8)	0.07550 (6)	0.43587 (16)	0.0279 (3)	
H13A	0.5313	0.0366	0.3930	0.034*	
H13B	0.5499	0.0718	0.5390	0.034*	
C14	0.64869 (9)	0.08217 (6)	0.39547 (17)	0.0321 (3)	
H14	0.6808	0.0442	0.4292	0.039*	
C15	0.68529 (9)	0.14227 (7)	0.46296 (18)	0.0358 (4)	
H15A	0.6804	0.1394	0.5663	0.043*	
H15B	0.7460	0.1463	0.4388	0.043*	
C16	0.65534 (9)	0.08664 (6)	0.23537 (17)	0.0341 (4)	
H16A	0.7156	0.0898	0.2075	0.041*	
H16B	0.6313	0.0479	0.1922	0.041*	
N1	0.28451 (7)	0.17669 (6)	0.44376 (15)	0.0376 (3)	
H1	0.2595	0.1786	0.5262	0.045*	
O1	−0.01378 (6)	0.03915 (5)	0.34139 (12)	0.0391 (3)	
O2	−0.02969 (6)	0.14408 (5)	0.32255 (14)	0.0420 (3)	
O3	0.27389 (7)	0.16496 (7)	0.21147 (14)	0.0595 (4)	
O4	0.37279 (6)	0.18202 (4)	0.43133 (12)	0.0349 (3)	
O5	0.37653 (7)	0.07535 (5)	0.43361 (14)	0.0506 (4)	

### Atomic displacement parameters (Å^2^)

**Table:**

	*U*^11^	*U*^22^	*U*^33^	*U*^12^	*U*^13^	*U*^23^
C1	0.0367 (8)	0.0359 (7)	0.0464 (10)	−0.0018 (6)	0.0100 (8)	−0.0004 (7)
C2	0.0327 (7)	0.0296 (6)	0.0223 (7)	0.0045 (5)	0.0017 (6)	−0.0017 (6)
C3	0.0298 (7)	0.0359 (7)	0.0384 (9)	0.0084 (6)	0.0016 (7)	−0.0012 (6)
C4	0.0256 (7)	0.0431 (8)	0.0497 (11)	0.0040 (6)	−0.0011 (7)	−0.0097 (7)
C5	0.0289 (7)	0.0449 (8)	0.0406 (10)	0.0021 (6)	0.0015 (8)	−0.0026 (7)
C6	0.0271 (7)	0.0301 (6)	0.0281 (8)	−0.0026 (5)	0.0018 (6)	0.0000 (6)
C7	0.0217 (6)	0.0227 (6)	0.0206 (7)	−0.0040 (4)	0.0008 (6)	0.0009 (5)
C8	0.0281 (7)	0.0299 (6)	0.0211 (7)	−0.0070 (5)	−0.0028 (6)	0.0010 (5)
C9	0.0313 (7)	0.0299 (6)	0.0203 (7)	−0.0075 (5)	0.0042 (6)	0.0012 (5)
C10	0.0273 (6)	0.0239 (6)	0.0318 (9)	−0.0060 (5)	0.0018 (6)	0.0058 (5)
C11	0.0270 (6)	0.0253 (6)	0.0289 (8)	−0.0078 (5)	−0.0055 (6)	−0.0005 (5)
C12	0.0305 (7)	0.0268 (6)	0.0234 (8)	−0.0031 (5)	−0.0018 (6)	−0.0027 (5)
C13	0.0306 (7)	0.0238 (6)	0.0294 (8)	−0.0035 (5)	0.0021 (7)	0.0074 (5)
C14	0.0264 (7)	0.0267 (6)	0.0433 (10)	0.0033 (5)	−0.0003 (7)	0.0103 (6)
C15	0.0259 (7)	0.0435 (8)	0.0382 (9)	−0.0039 (6)	−0.0099 (7)	0.0099 (7)
C16	0.0304 (7)	0.0259 (6)	0.0459 (10)	−0.0028 (5)	0.0123 (7)	−0.0021 (6)
N1	0.0215 (6)	0.0512 (7)	0.0401 (8)	0.0015 (5)	0.0037 (6)	−0.0063 (6)
O1	0.0342 (5)	0.0295 (5)	0.0538 (8)	0.0035 (4)	0.0041 (5)	−0.0012 (5)
O2	0.0312 (5)	0.0327 (5)	0.0621 (8)	0.0069 (4)	0.0012 (6)	0.0032 (5)
O3	0.0365 (7)	0.1062 (11)	0.0358 (8)	0.0027 (7)	0.0009 (6)	0.0004 (7)
O4	0.0213 (5)	0.0350 (5)	0.0485 (7)	0.0011 (4)	0.0008 (5)	−0.0027 (5)
O5	0.0314 (5)	0.0362 (6)	0.0841 (10)	−0.0085 (4)	0.0154 (6)	0.0045 (6)

### Geometric parameters (Å, °)

**Table:**

C1—O1	1.4498 (17)	C9—C16	1.5296 (19)
C1—H1A	0.9800	C9—C10	1.5316 (18)
C1—H1B	0.9800	C9—H9	1.0000
C1—H1C	0.9800	C10—C11	1.525 (2)
C2—O2	1.2005 (16)	C10—H10A	0.9900
C2—O1	1.3340 (16)	C10—H10B	0.9900
C2—C3	1.5038 (19)	C11—C15	1.5289 (19)
C3—C4	1.519 (2)	C11—C12	1.5364 (18)
C3—H3A	0.9900	C11—H11	1.0000
C3—H3B	0.9900	C12—H12A	0.9900
C4—C5	1.512 (2)	C12—H12B	0.9900
C4—H4A	0.9900	C13—C14	1.5327 (19)
C4—H4B	0.9900	C13—H13A	0.9900
C5—O3	1.230 (2)	C13—H13B	0.9900
C5—N1	1.322 (2)	C14—C16	1.533 (2)
C6—O5	1.1913 (16)	C14—C15	1.533 (2)
C6—O4	1.3792 (16)	C14—H14	1.0000
C6—C7	1.5090 (18)	C15—H15A	0.9900
C7—C13	1.5329 (17)	C15—H15B	0.9900
C7—C12	1.5443 (17)	C16—H16A	0.9900
C7—C8	1.5510 (19)	C16—H16B	0.9900
C8—C9	1.5308 (18)	N1—O4	1.4029 (14)
C8—H8A	0.9900	N1—H1	0.8800
C8—H8B	0.9900		
			
O1—C1—H1A	109.5	C11—C10—H10A	109.7
O1—C1—H1B	109.5	C9—C10—H10A	109.7
H1A—C1—H1B	109.5	C11—C10—H10B	109.7
O1—C1—H1C	109.5	C9—C10—H10B	109.7
H1A—C1—H1C	109.5	H10A—C10—H10B	108.2
H1B—C1—H1C	109.5	C10—C11—C15	109.78 (12)
O2—C2—O1	123.41 (13)	C10—C11—C12	109.42 (11)
O2—C2—C3	124.90 (13)	C15—C11—C12	109.55 (11)
O1—C2—C3	111.67 (11)	C10—C11—H11	109.4
C2—C3—C4	111.98 (11)	C15—C11—H11	109.4
C2—C3—H3A	109.2	C12—C11—H11	109.4
C4—C3—H3A	109.2	C11—C12—C7	109.24 (11)
C2—C3—H3B	109.2	C11—C12—H12A	109.8
C4—C3—H3B	109.2	C7—C12—H12A	109.8
H3A—C3—H3B	107.9	C11—C12—H12B	109.8
C5—C4—C3	111.57 (12)	C7—C12—H12B	109.8
C5—C4—H4A	109.3	H12A—C12—H12B	108.3
C3—C4—H4A	109.3	C7—C13—C14	109.65 (10)
C5—C4—H4B	109.3	C7—C13—H13A	109.7
C3—C4—H4B	109.3	C14—C13—H13A	109.7
H4A—C4—H4B	108.0	C7—C13—H13B	109.7
O3—C5—N1	122.19 (14)	C14—C13—H13B	109.7
O3—C5—C4	123.55 (15)	H13A—C13—H13B	108.2
N1—C5—C4	114.25 (15)	C13—C14—C16	108.77 (12)
O5—C6—O4	121.84 (12)	C13—C14—C15	109.46 (12)
O5—C6—C7	127.62 (12)	C16—C14—C15	109.96 (11)
O4—C6—C7	110.54 (10)	C13—C14—H14	109.5
C6—C7—C13	108.46 (10)	C16—C14—H14	109.5
C6—C7—C12	112.83 (11)	C15—C14—H14	109.5
C13—C7—C12	109.90 (11)	C11—C15—C14	109.49 (11)
C6—C7—C8	107.50 (11)	C11—C15—H15A	109.8
C13—C7—C8	109.47 (11)	C14—C15—H15A	109.8
C12—C7—C8	108.61 (10)	C11—C15—H15B	109.8
C9—C8—C7	108.95 (11)	C14—C15—H15B	109.8
C9—C8—H8A	109.9	H15A—C15—H15B	108.2
C7—C8—H8A	109.9	C9—C16—C14	109.75 (11)
C9—C8—H8B	109.9	C9—C16—H16A	109.7
C7—C8—H8B	109.9	C14—C16—H16A	109.7
H8A—C8—H8B	108.3	C9—C16—H16B	109.7
C16—C9—C8	109.49 (10)	C14—C16—H16B	109.7
C16—C9—C10	109.40 (12)	H16A—C16—H16B	108.2
C8—C9—C10	109.83 (11)	C5—N1—O4	117.73 (13)
C16—C9—H9	109.4	C5—N1—H1	121.1
C8—C9—H9	109.4	O4—N1—H1	121.1
C10—C9—H9	109.4	C2—O1—C1	116.27 (11)
C11—C10—C9	109.72 (10)	C6—O4—N1	113.40 (10)
			
O2—C2—C3—C4	17.1 (2)	C13—C7—C12—C11	58.97 (15)
O1—C2—C3—C4	−164.72 (13)	C8—C7—C12—C11	−60.76 (14)
C2—C3—C4—C5	−173.84 (14)	C6—C7—C13—C14	177.11 (12)
C3—C4—C5—O3	70.9 (2)	C12—C7—C13—C14	−59.12 (15)
C3—C4—C5—N1	−110.41 (16)	C8—C7—C13—C14	60.09 (14)
O5—C6—C7—C13	−26.1 (2)	C7—C13—C14—C16	−60.43 (14)
O4—C6—C7—C13	154.74 (12)	C7—C13—C14—C15	59.74 (16)
O5—C6—C7—C12	−148.06 (16)	C10—C11—C15—C14	−59.45 (15)
O4—C6—C7—C12	32.74 (16)	C12—C11—C15—C14	60.73 (16)
O5—C6—C7—C8	92.22 (18)	C13—C14—C15—C11	−60.58 (16)
O4—C6—C7—C8	−86.97 (13)	C16—C14—C15—C11	58.86 (15)
C6—C7—C8—C9	−177.12 (10)	C8—C9—C16—C14	−61.11 (14)
C13—C7—C8—C9	−59.50 (12)	C10—C9—C16—C14	59.30 (14)
C12—C7—C8—C9	60.50 (13)	C13—C14—C16—C9	60.84 (13)
C7—C8—C9—C16	59.84 (14)	C15—C14—C16—C9	−59.02 (14)
C7—C8—C9—C10	−60.31 (13)	O3—C5—N1—O4	1.1 (2)
C16—C9—C10—C11	−59.98 (14)	C4—C5—N1—O4	−177.63 (12)
C8—C9—C10—C11	60.21 (14)	O2—C2—O1—C1	1.0 (2)
C9—C10—C11—C15	60.21 (14)	C3—C2—O1—C1	−177.17 (13)
C9—C10—C11—C12	−60.05 (13)	O5—C6—O4—N1	−7.7 (2)
C10—C11—C12—C7	60.71 (13)	C7—C6—O4—N1	171.54 (11)
C15—C11—C12—C7	−59.69 (15)	C5—N1—O4—C6	−81.34 (16)
C6—C7—C12—C11	−179.84 (11)		

### Hydrogen-bond geometry (Å, °)

**Table:**

*D*—H···*A*	*D*—H	H···*A*	*D*···*A*	*D*—H···*A*
N1—H1···O3^i^	0.88	1.87	2.7250 (19)	165

Symmetry codes: (i) −*x*+1/2, *y*, *z*+1/2.
